# Discovery of Novel Acetylcholinesterase Inhibitors as Potential Candidates for the Treatment of Alzheimer’s Disease

**DOI:** 10.3390/ijms20041000

**Published:** 2019-02-25

**Authors:** Minky Son, Chanin Park, Shailima Rampogu, Amir Zeb, Keun Woo Lee

**Affiliations:** Division of Life Science, Division of Applied Life Science (BK21 Plus), Plant Molecular Biology and Biotechnology Research Center (PMBBRC), Research Institute of Natural Science (RINS), Gyeongsang National University (GNU), 501 Jinju-daero, Jinju 52828, Korea; minky@gnu.ac.kr (M.S.); chaninpark0806@gmail.com (C.P.); shailima.rampogu@gmail.com (S.R.); zebamir85@gmail.com (A.Z.)

**Keywords:** acetylcholinesterase, Alzheimer’s disease, molecular docking, molecular dynamics simulation, pharmacophore modeling

## Abstract

Acetylcholinesterase (AChE) catalyzes the hydrolysis of neurotransmitter acetylcholine to acetate and choline in a synaptic cleft. Deficits in cholinergic neurotransmitters are linked closely with the progression of Alzheimer’s disease (AD), which is a neurodegenerative disorder characterized by memory impairment, and a disordered cognitive function. Since the previously approved AChE inhibitors, donepezil (Aricept), galantamine (Reminyl), and rivastigmine (Exelon), have side effects and several studies are being carried out out to develop novel AD drugs, we have applied a three-dimensional quantitative structure−activity relationship (3D QSAR) and structure-based pharmacophore modeling methodologies to identify potential candidate inhibitors against AChE. Herein, 3D QSAR and structure-based pharmacophore models were built from known inhibitors and crystal structures of human AChE in complex with donepezil, galantamine, huperzine A, and huprine W, respectively. The generated models were used as 3D queries to screen new scaffolds from various chemical databases. The hit compounds obtained from the virtual screening were subjected to an assessment of drug-like properties, followed by molecular docking. The final hit compounds were selected based on binding modes and molecular interactions in the active site of the enzyme. Furthermore, molecular dynamics simulations for AChE in complex with the final hits were performed to evaluate that they maintained stable interactions with the active site residues. The binding free energies of the final hits were also calculated using molecular mechanics/Poisson-Boltzmann surface area method. Taken together, we proposed that these hits can be promising candidates for anti-AD drugs.

## 1. Introduction

Alzheimer’s disease (AD) is a neurodegenerative disorder that is characterized by multiple cognitive impairments such as memory loss and difficulties in learning and/or thinking. It has been investigated that the formation of cortical amyloid plaques and neurofibrillary tangles in the brain are the fundamental hallmarks of AD patients. Furthermore, AD is closely related with neurotransmitter acetylcholine deficiency in the hippocampus and cerebral cortex [1,2]. The hydrolysis of acetylcholine to acetate and choline is catalyzed by acetylcholinesterase (AChE) in a synaptic cleft. Currently, AChE inhibitors including donepezil (Aricept), galantamine (Reminyl), and rivastigmine (Exelon), are widely used in symptomatic treatments for AD [3,4,5,6]. But the efficacy of these drugs in hampered by their side effects, such as gastrointestinal disturbance, hepatotoxicity, and hypotension [7,8,9,10,11]. Therefore, inhibition of AChE still remains a promising strategy in AD management [12,13,14,15].

The structure of human AChE (hAChE) consists of a central 12-stranded mixed β-sheet surrounded by 14 α-helices. The active site of the enzyme is located near the bottom of a 20 Ǻ deep narrow gorge and is formed by a catalytic anionic site (CAS) containing a catalytic triad of Ser203, Glu334, and His447. The other key residues such as Asp74, Tyr124, Ser125, Trp286, Tyr337, and Tyr341 compose a peripheral anionic subsite (PAS) which is placed at the entrance of the active site gorge. In addition, other functional subsites, known as anionic subsite (AS), acyl-binding pocket (ABP), and oxyanion hole (OH), found in an active site gorge, are also reported to play important roles in the recognition process of the enzyme. In this study, we have employed a three-dimensional quantitative structure−activity relationship (3D QSAR) and structure-based pharmacophore modeling approach in order to discover potential candidates of hAChE inhibitors. The generated pharmacophore models were used for screening chemical databases, and then the obtained hit compounds were filtered by drug-like property evaluation. The binding mode analyses for hit compounds were performed by utilizing molecular docking and molecular dynamics (MD) simulation studies. The binding free energy between the protein and the compound was calculated using molecular mechanics/Poisson-Boltzmann surface area (MM-PBSA) method.

## 2. Results and Discussion

### 2.1. Generation of 3D QSAR Pharmacophore Model

**Figure 1:**
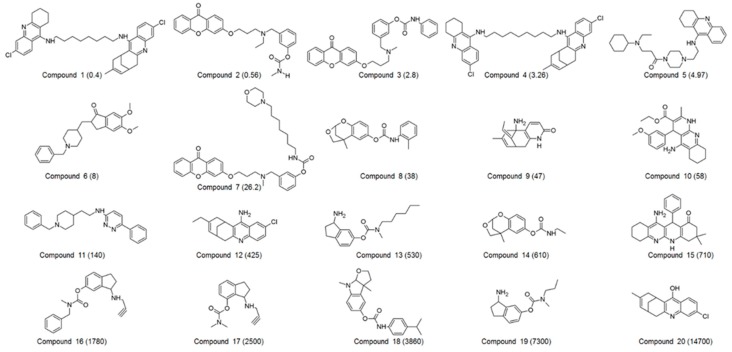
2D structures of 20 compounds in the training set. The inhibitory activity value (IC_50_) for each compound was shown in nM.

A set of 60 compounds with diverse structural scaffolds were prepared for 3D QSAR pharmacophore modeling. Their inhibitory activities ranged from 0.065 to 15,700 nM. Among 60 compounds, 20 compounds were selected as a training set, which was used for the generation of a 3D QSAR pharmacophore model. The 2D structures and IC_50_ values of the training set were shown in Figure 1.

The remaining 40 compounds were considered a test set which was used to validate the model (Figure S1). All compounds in training and test sets were classified into four groups based on their IC_50_ values: most active (IC_50_ < 20 nM), active (20 ≤ IC_50_ < 200 nM), moderately active (200 ≤ IC_50_ < 2000 nM), and inactive (IC_50_ ≥ 2000 nM). A set of 10 hypotheses were constructed using a training set of 20 compounds. The statistical parameters of the top 10 hypotheses were listed in Table 1. As shown in Table 1, the null cost and fixed cost were 215.87 and 79.29, respectively. The cost analyses showed that Hypo (hypothesis) 1 and 2 have the largest cost difference of 116.592, signifying the highest predictive power. 

**Figure 2:**
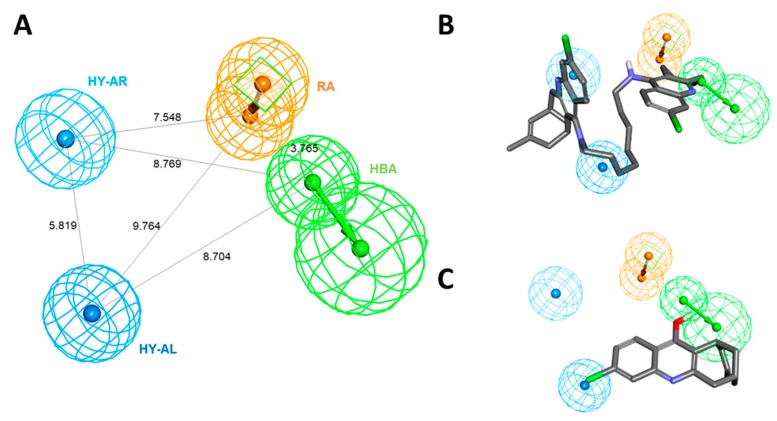
The best hypothesis, Hypo 1, with their distance constraints. (**A**) Hypo 1 consists of four pharmacophoric features such as HBA (green), HY-AL (blue), HY-AR (cyan), and RA (orange). (**B**) Hypo 1 overlay onto most active compound, compound 1 (IC_50_ = 0.4 nM) from the training set. (**C**) Hypo 1 overlay onto inactive compound, compound 20 (IC_50_ = 14700 nM) from the training set.

Among the generated hypotheses, Hypo 1 (named as Pharm 1) was selected as the best hypothesis due to the highest cost difference, lowest total cost, lowest RMSD, and highest correlation coefficient. Our results demonstrated that Pharm 1 consists of four pharmacophoric features including hydrogen bond acceptor (HBA), hydrophobic aliphatic (HY-AL), hydrophobic aromatic (HY-AR), and ring aromatic (RA) (Figure 2A). The most active and inactive compounds in the training set were aligned to the pharmacophore model. The compound 1, one of the most active compounds, was well fitted into all chemical features in Pharm 1 while compound 20, the inactive compound, was mapped only onto two of four features (Figure 2B,C).

To verify the predictive ability of the model, the activities of training set compounds were estimated using regression analysis. The experimental and estimated activity values for each compound in the training set were shown in Table 2.

**Figure 3:**
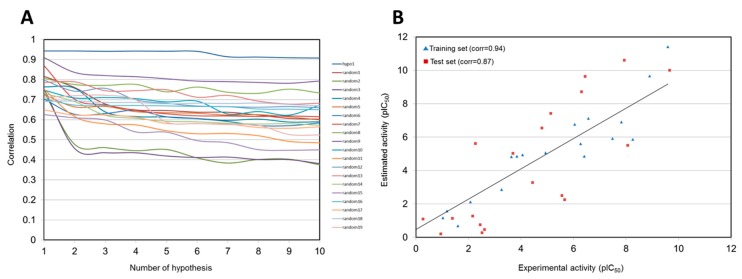
Validation of Hypo 1 model. (**A**) Comparison of the correlations for Hypo 1 and 19 random spreadsheets in Fischer’s randomization test. (**B**) Correlations between the experimental and predicted activities for the training set and test set against Hypo 1.

As a result, 17 out of 20 training set compounds showed that the predicted activity scales were the same as those of their experimental activity scale. Only one moderately active compound was predicted as active, and two inactive compounds were classified into moderately actives. Pharm 1 was subsequently validated using Fischer’s randomization and test set methods. In Fischer’s randomization test, 19 random spreadsheets for training set compounds were generated. At 95% confidence level, Pharm 1 showed the highest correlation of all the random spreadsheets (Figure 3A). This result indicated that Pharm 1 was not generated by chance. For test set validation, the predicted activities for 40 compounds in the test set were calculated with the same procedures as used in the training set (Table S1). The plot showed that correlation coefficient between the experimental and predicted activity scales is 0.87 in the test set, confirming the statistical significance of Pharm 1 (Figure 3B).

### 2.2. Development of Structure-Based Pharmacophore Models

**Figure 4:**
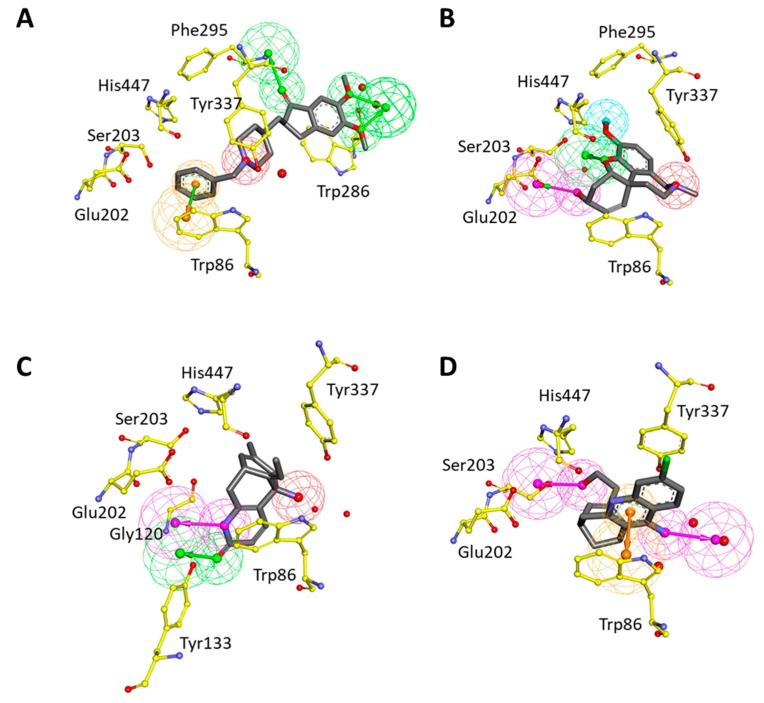
Structure-based pharmacophore models from hAChE-inhibitor complexes. Each model was generated from (**A**) donepezil (DNP), (**B**) galantamine (GNT), (**C**) huperzine A (HUP), and (**D**) huprine W (HUW) bound structure, respectively. The interacting residues of hAChE were displayed in yellow stick models. The pharmacophoric features, HBA (green), HY-AL (blue), HY-AR (cyan), RA (orange), and PI (red) were shown.

Structure-based pharmacophore models were generated using four complex structures with different inhibitors (DNP, GNT, HUP, and HUW) of hAChE. During hypotheses generation, water molecules in the crystal structures were included to consider water-mediated hydrogen bond interactions. For each structure, the best pharmacophore model was selected based on the selectivity score. The best hypothesis (Pharm 2), which was built by DNP-bound structure, has three HBA, one PI, and one RA feature (Figure 4A). HBA features were obtained based on hydrogen bond interactions with water molecules and Phe295. The PI feature represented the interaction point near Tyr337 to harbor the positively ionizable group. The RA feature was matched with π–π interaction between DNP and Trp86. In GNT-bound structure, the best hypothesis (Pharm 3) was comprised of four pharmacophoric features such as one HBA, one HBD, one HY, and one PI (Figure 4B). HBA and HBD features were represented by hydrogen bond interactions with a water molecule, Glu202, and Ser203. HY and PI features corresponded with the hydrophobic and positive ionizable sites close to Phe295 and Tyr337, respectively. The hypothesis (Pharm 4) generated from HUP-bound structure consisted of one HBA, one HBD, and one PI features (Figure 4C). HBA and HBD features represented hydrogen bonds with Tyr133 and a water molecule, respectively. PI feature accounted for the interaction point surrounded by two water molecules, Trp86 and Tyr337. The last hypothesis (Pharm 5), constructed from the HUW-bound structure has two HBD and one RA feature (Figure 4D). The one HBD feature was generated from a hydrogen bond interaction with Ser203 while another was from an interaction with the water molecule. The RA feature reflected π–π interaction between HUW and Trp86. As a result of the structure-based pharmacophore modeling, it was observed that most of the hypotheses share the pharmacophoric features generated from Trp86, Ser203, Phe295, and Tyr337. This implicated that molecular interactions with these residues were important for inhibitor binding to the active site of hAChE.

### 2.3. Identification of Candidate Hits from Database Screening and Molecular Docking

**Figure 5:**
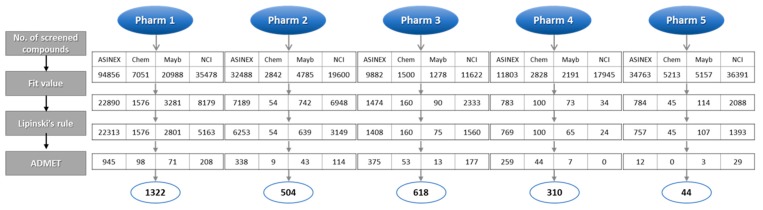
Database screening using pharmacophore models (Pharm 1–5). The number of hit compounds in each filtration process was shown along with the database name.

All five pharmacophore models (Pharm 1 to 5) were used as 3D queries to screen hundreds of thousands of chemical compounds. The databases used for the screening were ASINEX, Chembridge, Maybridge, and NCI that contain 213,262, 50,000, 59,652, and 238,819 compounds, respectively. Initially, compounds that matched all of the features of each model were screened and then further selected based on a fit value. For the screened compounds from Pharm 1, the fit value of 8, which was greater than maximum fit value of the training set, was used. In Pharm 2 to 5, the fit value from each reference compound used to build the pharmacophore model was used. The next filtration was done by applying Lipinski’s rule of five and ADMET properties in order to exclude non drug-like compounds. The selected hit compounds which were specially predicted to have high Blood–Brain Barrier (BBB) penetration ability were subjected to molecular docking. The number of hit compounds in each step was shown in Figure 5 in accordance with database name.

The hit compounds retrieved from Pharm 1 and the training set were docked into DNP-bound structures while the compounds from Pharm 2, 3, 4, and 5 were docked to their corresponding crystal structure along with their co-crystal ligands. The GOLD fitness scores of each reference compound were 48.38, 64.89, 64.81, and 41.70 for DNP in Pharm2, GNT in Pharm 3, HUP in Pharm 4, and HUW in Pharm 5, respectively. The docked pose of each compound showed identical conformation to that in the experimental one, signifying this docking approach can reproduce the experimentally determined binding mode of the known AChE inhibitors. As a result of Pharm 1544 hit compounds which have a higher GOLD fitness score than that of the most active compound in the training set were selected. Subsequently, 4 hits compounds were chosen based on visual inspection and their interactions with key residues of hAChE. Thereafter, hit compounds derived from Pharm 2 to 5 were also analyzed in the same manner. Finally, 4, 2, 3, 2 hit compounds were selected from Pharm 2, 3, 4, and 5, respectively. At the end, 15 hit compounds were further evaluated using MD simulation.

### 2.4. Selection of Hit Compounds and Their Binding Modes at the active Site of hAChE

**Figure 6:**
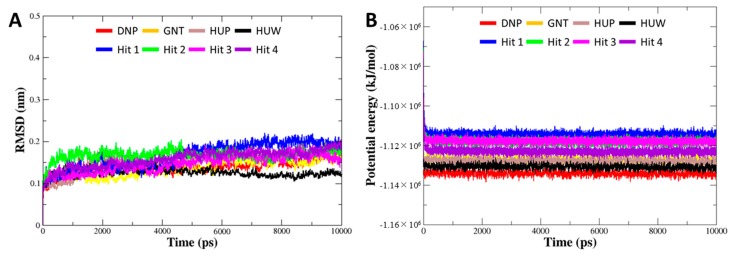
Overall stability of MD trajectory of each system. (**A**) Root mean square deviation (RMSD) and (**B**) potential energy for the system were calculated during 10 ns simulation time.

To investigate the binding stability of each hit compound at the active site of hAChE, MD simulation was performed. Our results identified that four hit compounds showed consistent interactions with the active site residues of hAChE. The C_α_ RMSD and potential energy for 8 systems (four of each reference and final hit) were computed to probe into overall stability of the simulations. The RMSD values for all of the systems were converged to less than 0.2 nm (Figure 6A). The potential energies were also well equilibrated, demonstrating that all of the simulations remained stable without any abnormal behavior in the structures during the entire simulation period (Figure 6B). The binding modes for final hits were analyzed using the representative structure with the lowest potential energy. The structural alignment of all the reference and hit compounds suggested that each hit compound occupied the active site of hAChE in a similar pattern as the reference compounds.

**Figure 7:**
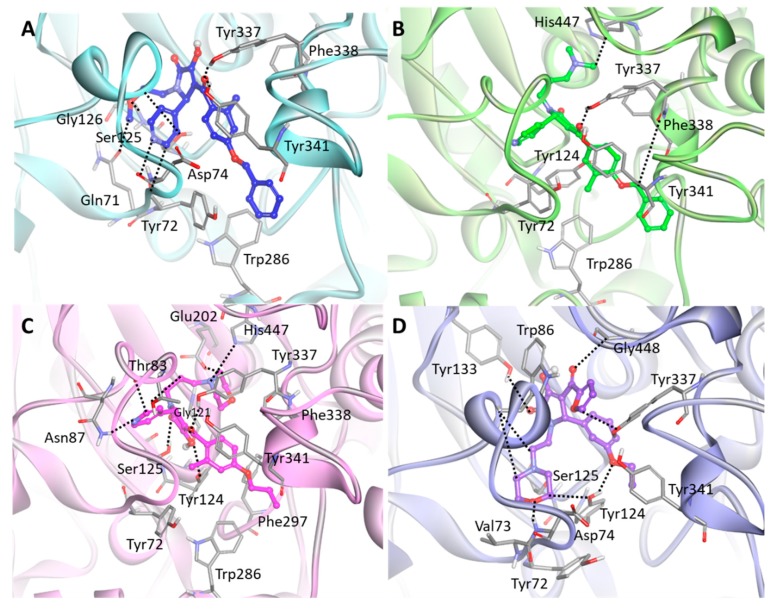
Binding mode of the final hit compounds at the active site of hAChE. (**A**) Hit 1 (**B**) Hit 2 (**C**) Hit 3 (**D**) Hit 4. Each hit compound was drawn as blue, green, magenta, and purple stick model. The interacting residues are shown as gray stick models. Hydrogen bonds between hAChE and the compound are indicated as black dashed lines. Only polar hydrogens are shown for clarity.

The detailed molecular interaction analyses of Hit 1 revealed that the compound formed hydrogen bond interactions with PAS residues of hAChE (Figure 7A). The pyridine ring established hydrogen bonds with Tyr72 and Asp74. The methoxyethyl group displayed hydrogen bonding to Gln71, Ser125, and Gly126, while the carbonyl group between methylbenzene and pyrrole moieties showed hydrogen bond interactions with Tyr337 and Tyr341. The benzene ring of the compound interacted with the aromatic rings of Tyr72 and Trp286 through π–π stacking. Also, the methylbenzene moiety formed π–π stacking and π-alkyl interaction with Phe338 and Tyr341, respectively. The binding of Hit 2 was stabilized by molecular interactions with several key residues at the PAS site including Tyr72, Tyr124, Trp286, and Tyr341 (Figure 7B). The compound formed hydrogen bond interactions with Tyr337, Phe338, and catalytic triad His447. Also, the methylbenzene moiety of the compound formed π–π stacking to the aromatic ring of Tyr341 while the methyl group of the methylbenzene moiety made π-alkyl interactions with Tyr72, Tyr124, and Trp286. In Hit 3, hydrogen bonds between the compound and the active site residues such as Thr83, Asn87, Gly121, Tyr124, Ser125, Glu202, Tyr337, and His447 were observed (Figure 7C). The methylbenzene moiety of the compound located between Tyr124 and Tyr341 by forming π–π T-shaped and π–π stacking interactions with their aromatic rings. Additionally, the methyl group of the methylbenzene moiety formed π-alkyl interaction with Tyr72. The morpholine ring moiety buried at the catalytic triad was found to have π-alkyl interactions with Phe338 and His447, while dimethyl moiety exposed to ABP site had π-alkyl interactions with Trp286 and Phe297. The binding mode of Hit 4 showed that almost all residues in PAS and AS sites such as Tyr72, Asp74, Trp86, Tyr124, Ser125, Tyr133, Tyr337, and Gly448 were involved in hydrogen bond interactions with the compound (Figure 7D). The furan and benzene ring moieties of the compound established π–π stacking and π–π T-shaped interactions with the aromatic rings of Trp86 and Tyr337, respectively. The morpholine ring and methyl group at both ends of the compound formed π-alkyl interactions with Val73, Tyr124, and Tyr341.

**Figure 8:**
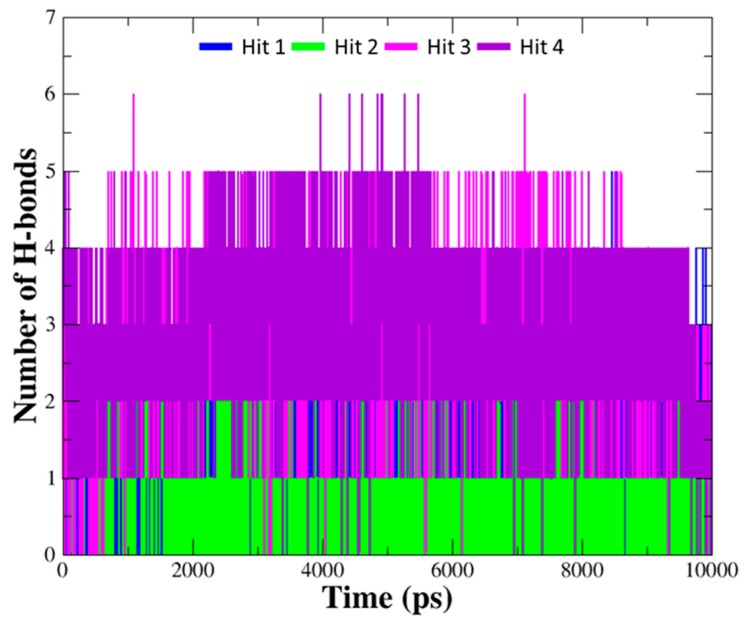
The number of hydrogen bonds between hAChE and hit compounds. Hydrogen bond interactions for each hit compound were monitored during10 ns simulation time. The number of hydrogen bonds for each hit compound are indicated as blue, green, magenta, and purple lines.

The details of the molecular interactions between each compound and hAChE were summarized in Table 3. When compared to interaction of the reference compounds, the hits establish diverse interaction networks with active site residues as well as encompass the interactions with important residues including Trp86, Ser203, Phe295, and Tyr337 found in the reference compounds. Moreover, the number of hydrogen bonds between hit compounds and hAChE was monitored during the entire simulation time (Figure 8). The average number of hydrogen bonds were 1.37, 1.04, 2.79, and 2.91 for Hit 1, Hit 2, Hit 3, and Hit 4, which is more than that of the reference compounds (0.91, 1.48, 1.09, 0.96 for DNP, GNT, HUP, HUW). It was observed that all of the hits formed stable hydrogen bond interactions with the enzyme.

Binding free energies between the compounds and hAChE were predicted using MM-PBSA method. Average binding free energies for reference compounds ranged from −172.86 kJ/mol to −105.98 kJ/mol (Table 4). Average values for hit compounds were −147.77 kJ/mol, −165.51 kJ/mol, −172.80 kJ/mol, and −146.96 kJ/mol for Hit 1, Hit 2, Hit 3, and Hit 4, respectively.

**Figure 9:**
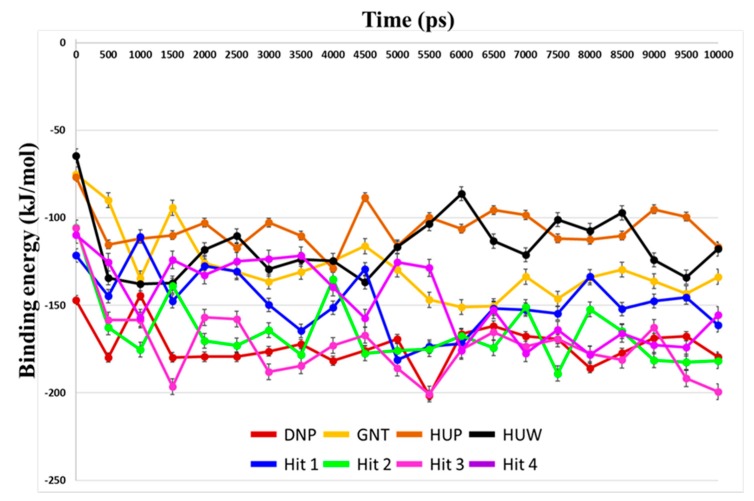
Prediction of binding free energies by MM-PBSA calculation. Binding free energy between hAChE and each reference and hit compounds was calculated using MD trajectories. Energy values for DNP, GNT, HUP, HUW, Hit 1, Hit 2, Hit3, and Hit4 are shown as red, yellow, brown, black, blue, green, magenta, and purple lines, respectively.

**Figure 10:**
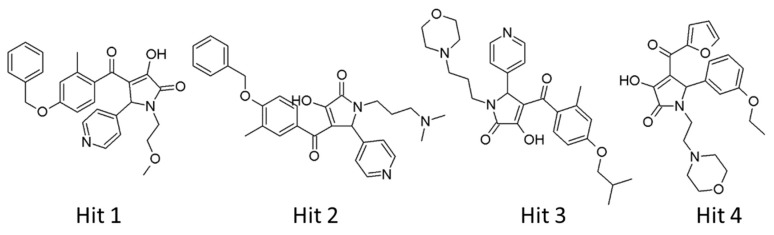
2D structures of final hit compounds.

Our analyses showed that overall binding free energies for hit compounds were lower than those of the reference compounds, revealing that the binding of the selected hit compounds to hAChE were favorable (Figure 9). Finally, these hit compounds were suggested as novel hAChE inhibitors, and their 2D structures have been shown in Figure 10. Also, their physico-chemical properties were shown in Table S2.

## 3. Materials and Methods

### 3.1. D QSAR Pharmacophore Modeling

A total of 60 compounds were collected from the literatures and the bindingDB database [16,17,18,19,20,21,22,23,24,25,26,27,28,29,30,31,32,33,34,35]. All compounds have the inhibitory activities (IC_50_) against hAChE which were determined under the same biological assay conditions. The compounds were divided into training set and test set. A training set of structurally diverse 20 compounds were used to build 3D QSAR pharmacophore hypotheses. The 3D structures of the compounds were relaxed to the nearest local minimum by energy minimization (EM). The EM with CHARMm force field was performed using Minimize Ligands protocol with smart minimizer algorithm for 2000 steps implemented in Discovery Studio (DS) 2016 (BIOVIA, San Diego, CA, USA).

Ligand-based pharmacophore modeling was carried out by 3D QSAR Pharmacophore Generation protocol with HypoGen algorithm in DS. Prior to model generation, the low energy conformations were prepared by enabling the Conformation Generation option with BEST algorithm. The uncertainty values for training set compounds were set to 2 or 3 while other parameters were used as defaults. For hypotheses generation, pharmacophoric features such as hydrogen bond acceptor (HBA), hydrogen bond donor (HBD), ring aromatic (RA), negative ionizable (NI), positive ionizable (PI), and hydrophobic (HY) features including hydrophobic aliphatic (HY-AL) and hydrophobic aromatic (HY-AR) were considered. The generated hypotheses were ranked by statistical parameters, which consist of null cost, total cost, fixed cost, root mean square deviation (RMSD), and correlation (*r*). The significance of the hypotheses were assessed based on their statistical parameters by Debnath’s method [36].

The best hypothesis was validated using Fischer’s randomization and test set methods. In Fischer’s randomization test, the CatScramble program produces a set of 19 random spreadsheets generating 10 hypotheses in each run and then calculates total cost value or correlation between the chemical structures and the biological activity. A confidence level of 95% was used during the Fischer’s randomization test. The hypothesis is considered to be generated by chance if any of the randomly generated hypotheses showed better total cost or correlation than the best hypothesis. In the test set validation, 40 test set compounds were used to verify whether the hypothesis was able to predict the activity values and to classify the compounds into their experimental activity ranges. The validation was performed using Ligand Pharmacophore Mapping protocol with FAST and Flexible search options in DS. The low energy conformations of the test set compounds were generated by the same procedures used for the training set.

### 3.2. Structure-Based Pharmacophore Modeling

The X-ray crystal structures of hAChE in complex with four different inhibitors, donepezil (DNP, PDB ID: 4EY7), galantamine (GNT, 4EY6), huperzine A (HUP, 4EY5), and huprine W (HUW, 4BDT), were obtained from RCSB Protein Data Bank (http://www.rcsb.org; accessed on 15 December 2018) to generate structure-based pharmacophore models [37,38]. Missing regions in the structures were recovered using Prepare Protein tool in DS. All other co-crystal ligands were removed. Structure-based pharmacophore modeling was performed by Receptor-ligand pharmacophore generation protocol of DS. This module predicts pharmacophoric features based on molecular interactions between the active site residues of hAChE and the bound inhibitor. To consider the flexibility of protein, Maximum hydrogen bond distance was changed from 3.0 to 3.5 Å and all other parameters were kept as default values. The generated hypotheses were ranked by the selectivity score. Higher score implies that the corresponding hypothesis has greater potential of target selectivity. Finally, the hypothesis with highest selectivity score was selected as the best model.

### 3.3. Pharmacophore-Based Database Screening

Virtual screening of chemical databases was executed by Ligand Pharmacophore Mapping protocol available in DS. During the database screening, the parameters were the same as those in 3D QSAR pharmacophore modeling procedure, except that Conformation Generation option was changed to FAST algorithm to reduce computational cost. The screened compounds were sequentially filtered by evaluating drug-like properties such as Lipinski’s rule of five [39] and absorption, distribution, metabolism, excretion, and toxicity (ADMET) [40]. The compounds passing all criteria were subjected to molecular docking calculation.

### 3.4. Molecular Docking

Molecular docking of compounds into the active site of hAChE was performed through Genetic Optimization for Ligand Docking (GOLD v5.2.2, The Cambridge Crystallographic Data Centre, Cambridge, UK). GOLD is an automated docking software to predict the ligand conformational flexibility by genetic algorithm [41,42]. The geometries of compounds were minimized using the same protocol as described previously. The protonation states of all titratable residues in the protein were set to pH 7.0 by Clean Protein tool implemented in DS. All atoms within 10 Å in the vicinity of the bound inhibitor were defined as the binding site using the Define and Edit Binding Sites tool of DS. The number of docking runs was set to 10. The docking poses were ranked by GOLD fitness score. The most populated conformation with high score was selected as the best pose of the compound. Final conformation was used as an initial structure for MD simulation.

### 3.5. Molecular Dynamics Simulation

MD simulation of protein-ligand complex was conducted with AMBER03 force field [43] using GROMACS 5.1.4 package (GROningen MAchine for Chemical Simulations, www.gromacs.org). To generate the topology file for a ligand, AnteChamber Python Parser interface (ACPYPE) was used [44]. The structure was inserted into dodecahedron box of TIP3P water model [45]. Periodic boundary conditions were applied to avoid the edge effect. The system was neutralized by replacing water molecules with counter-ions. EM procedure with steepest descent algorithm was performed until the maximum force was converged to less than 1000 kJ/mol. The system was then equilibrated during 100 ps under NVT ensemble. Subsequently, 100 ps of NPT equilibration was executed. After two phases of equilibration procedures, 10 ns production run was performed under NPT ensemble. A constant temperature of 300 K and a pressure of 1 bar was maintained through Nose-Hoover thermostat [46,47] and Parrinello-Rahman barostat [48,49], respectively. All bond lengths were restrained by LINCS algorithm [50,51]. The water molecules were constrained by SETTLE algorithm [52]. The particle mesh Ewald method [53,54] was used to compute long-range electrostatic interactions. Cut-off values for calculating short-range electrostatic and van der Waals interactions were 1.2 nm. The time step of the simulation was 2 fs, and the atomic coordinates were saved every 1 ps.

### 3.6. Binding Free Energy Calculation

The MM-PBSA binding free energy between protein and ligand was calculated using the g_mmpbsa tool [55] of GROMACS. This tool computes molecular mechanics potential energy which is comprised of electrostatic and van der Waals interactions and solvation free energy, including polar and nonpolar solvation energies. The entropic contribution was not considered in this method. The binding free energy calculation was performed using 20 snapshots which were sampled every 500 ps from the trajectory of the 10 ns MD simulation, using the default parameters.

## 4. Conclusions

In our current study, we have developed one 3D QSAR pharmacophore model from structurally diverse hAChE inhibitors and four structure-based pharmacophore models from hAChE structure in complex with donepezil, galantamine, huperzine A, and huprine W. The validated best models were used as 3D queries to screen ASINEX, Chembridge, Maybridge, and NCI chemical databases. Subsequently, the retrieved compounds were filtered through drug-like properties evaluation and molecular docking calculations. The binding of hit candidates with hAChE were assessed by MD simulations and binding free energies calculation. Finally, four hit compounds which formed favorable interactions at the active site of the enzyme were proposed as potential candidate molecules against hAChE. Furthermore, our results might be helpful in designing novel inhibitors of AChE to be used for the treatment of AD.

## Figures and Tables

**Table 1:** 3D QSAR pharmacophore generation. Ten hypotheses were listed with their statistical parameters.

Hypothesis	Total Cost	Cost Difference ^a^	RMSD ^b^	Correlation	Features ^c^
Hypo 1	99.280	116.592	1.323	0.943	HBA, HY-AL, HY-AR, RA
Hypo 2	99.280	116.592	1.324	0.943	HBA, HY-AL, HY-AR, RA
Hypo 3	99.681	116.191	1.343	0.941	HBA, HY-AL, HY-AR, RA
Hypo 4	99.751	116.121	1.331	0.942	HBA, HY-AL, HY-AR, RA
Hypo 5	99.957	115.915	1.340	0.942	HBA, HY-AL, HY-AR
Hypo 6	100.012	115.860	1.346	0.941	HBA, HY-AL, 2HY-AR
Hypo 7	105.818	110.054	1.606	0.914	HBA, HY-AL, HY-AR, RA
Hypo 8	106.957	108.915	1.620	0.913	HBA, HY-AL, HY-AR, RA
Hypo 9	108.050	107.822	1.650	0.908	HBA, HY-AL, HY-AR, RA
Hypo 10	108.40	107.474	1.663	0.908	HBA, HY-AL, HY-AR, RA

^a^ Cost Difference = Null cost − Total cost. Null cost = 215.87, Fixed cost = 79.29 (All cost units are in bits). ^b^ RMSD = Root Mean Square Deviation. ^c^ Features = HBA, hydrogen bond acceptor; HY-AL, hydrophobic aliphatic; HY-AR, hydrophobic aromatic; RA, ring aromatic.

**Table 2:** Experimental and estimated IC_50_ values of the training set using the best hypothesis, Hypo 1.

Compound No.	Experimental IC_50_ (nM)	Estimated IC_50_ (nM)	Error ^a^	Experimental Scale ^b^	Estimated Scale ^b^	Fit Value ^c^
1	0.4	1.5	+3.8	++++	++++	7.21
2	0.56	0.93	+1.7	++++	++++	7.42
3	2.8	3.2	+1.1	++++	++++	6.89
4	3.26	4.7	+1.4	++++	++++	6.72
5	4.97	2.2	−2.3	++++	++++	7.05
6	8	8.9	+1.1	++++	++++	6.44
7	26.2	20	−1.3	+++	+++	6.08
8	38	120	+3.2	+++	+++	5.30
9	47	130	+2.7	+++	+++	5.29
10	58	140	+2.3	+++	+++	5.26
11	140	150	+1.1	+++	+++	5.21
12	425	850	+2	++	++	4.46
13	530	260	−2	++	++	4.98
14	610	130	−4.7	++	+++	5.28
15	710	510	−1.4	++	++	4.68
16	1780	360	−4.9	++	++	4.83
17	2500	980	−2.6	+	++	4.40
18	3860	320	−12	+	++	4.88
19	7300	15,000	+2	+	+	3.23
20	14,700	89,000	+6.1	+	+	2.44

^a^ Error: Difference between the experimental and estimated IC_50_ values. Positive value indicates that the estimated value is higher than the experimental value; negative value indicates that the estimated value is lower than the experimental value. ^b^ Activity scale: ++++, IC_50_ < 20 nM (most active); +++, 20 ≤ IC_50_ < 200 nM (active); ++, 200 ≤ IC_50_ < 2000nM (moderately active); +, IC_50_ ≥ 2000 nM (inactive). ^c^ Fit value represents how well the pharmacophoric features in the hypothesis overlap the chemical features in the compound.

**Table 3:** Molecular interactions between hAChE and the final hit compounds.

No.	Compound	Hydrogen Bond (Ǻ)	π–π Stacking	π-Alkyl	Hydrophobic
1	Hit 1	H56—Gln71:OE (2.53); H48—Tyr72:O (2.66); N15—Asp74:H (2.47); H39—Ser125:OG (2.49); O23—Gly126:HA2 (2.73); O11—Tyr337:HH (1.88); O11—Tyr341:HH (2.83)	Tyr72, Trp286, Tyr341	Phe338	Val73, Trp86, Pro88, Gly121, Tyr124, Ala127, Leu130, Tyr133, Glu202, Glu292, Ser293, Gly342, Gly448
2	Hit 2	O13—Tyr337:HH (1.87); H46—Phe338:O (2.70); H61—His447:O (2.47)	Tyr341	Tyr72, Tyr124, Trp286	Asp74, Thr83, Trp86, Asn87, Ser125, Tyr133, Glu202, Glu292, Ser293, Val294, Phe295, Phe297, Leu339, Gly342, Ala343, Gly448, Ile451
3	Hit 3	H58—Thr83:O (2.38); N16—Asn87:HD21 (2.74); H40—Gly121:O (3.05); O10—Gly121:HA (2.51); O15—Tyr124:HH (2.56); O10—Ser125:HG (2.25); H40—Ser125:OG (1.81); H60—Glu202:OE2 (2.71); O12—Tyr337:HH (2.16); H52—His447:NE2 (2.55); H53—His447:NE2 (2.72)	Tyr124, Tyr341	Tyr72, Trp286, Phe297, Phe338	Asp74, Trp86, Pro88, Gly122, Gly126, Ser203 Glu292, Ser293, Val294, Phe295, Arg296, Gly342
4	Hit 4	H50—Tyr72:O (2.02); O20—Asp74:H (2.72); H41—Trp86:O (2.97); H46—Trp86:O (2.94); O23—Tyr124:HH (2.41); H44—Tyr124:OH (2.99); N10—Ser125:HG (1.94); O11—Tyr133:HH (2.85); 17—Tyr337:HH (2.92); O14—Gly448:HA1 (2.84)	Trp86, Tyr337	Val73, Tyr124, Tyr341	Gln71, Gly82, Thr83, Asn87, Pro88, Gly120, Gly121, Gly122, Gly126, Leu130, Ser203, Phe297, Phe338, Trp439, His447

**Table 4:** Binding free energy (kJ/mol) calculated by MM-PBSA method. ΔE*_elec_*, electrostatic energy; ΔE*_vdw_*, van der Waal energy; ΔG*_polar_*, polar solvation energy; ΔG*_nonpolar_*, nonpolar solvation energy; ΔG*_binding_*, binding energy.

No.	Compound	ΔE*_vdw_*	ΔE*_elec_*	ΔG*_polar_*	ΔG*_nonpolar_*	ΔG*_binding_*
1	DNP	−229.18 ± 11.96	−25.55 ± 6.77	104.57 ± 15.24	−22.69 ± 0.89	−172.86 ± 12.06
2	GNT	−176.69 ± 8.24	−52.49 ± 18.03	116.92 ± 26.18	−16.00 ± 0.66	−128.25 ± 19.27
3	HUP	−174.90 ± 6.68	−37.84 ± 9.05	121.42 ± 15.04	−14.65 ± 0.65	−105.98 ± 11.26
4	HUW	−169.03 ± 13.95	−19.49 ± 15.20	89.95 ± 31.79	−17.54 ± 0.90	−116.11 ± 17.99
5	Hit 1	−246.59 ± 10.98	−26.22 ± 7.66	149.92 ± 18.92	−24.87 ± 0.99	−147.77 ± 17.30
6	Hit 2	−263.92 ± 14.68	−12.06 ± 9.93	137.80 ± 23.00	−27.32 ± 0.80	−165.51 ± 19.17
7	Hit 3	−273.73 ± 15.13	−51.68 ± 11.30	179.79 ± 24.75	−27.18 ± 0.94	−172.80 ± 20.28
8	Hit 4	−251.71 ± 16.46	−62.46 ± 15.70	190.82 ± 27.06	−23.61 ± 0.76	−146.96 ± 22.08

## Supplementary Materials

Supplementary materials can be found at https://www.mdpi.com/1422-0067/20/4/1000/s1.
