# Supplementary material for: Discovery of Novel Acetylcholinesterase Inhibitors as Potential Candidates for the Treatment of Alzheimer’s Disease
Source: Int J Mol Sci. 2019 Feb 25;20(4):1000. doi: 10.3390/ijms20041000 (PMC6412560; doi:10.3390/ijms20041000)
Supplement: Supplementary file 1 [file ijms-20-01000-s001.pdf]

Discovery of Novel Acetylcholinesterase Inhibitors as Potential Candidates for the Treatment of Alzheimer's Disease

Minky Son, Chanin Park, Shailima Rampogu, Amir Zeb, and Keun Woo Lee *

Division of Life Science, Division of Applied Life Science (BK21 Plus), Plant Molecular Biology and Biotechnology Research Center (PMBBRC), Research Institute of Natural Science (RINS), Gyeongsang National University (GNU), 501 Jinju-daero, Jinju 52828, Korea

* Corresponding author: kwlee@gnu.ac.kr; Tel: +82-55-772-1360, Fax: +82-55-772-1359

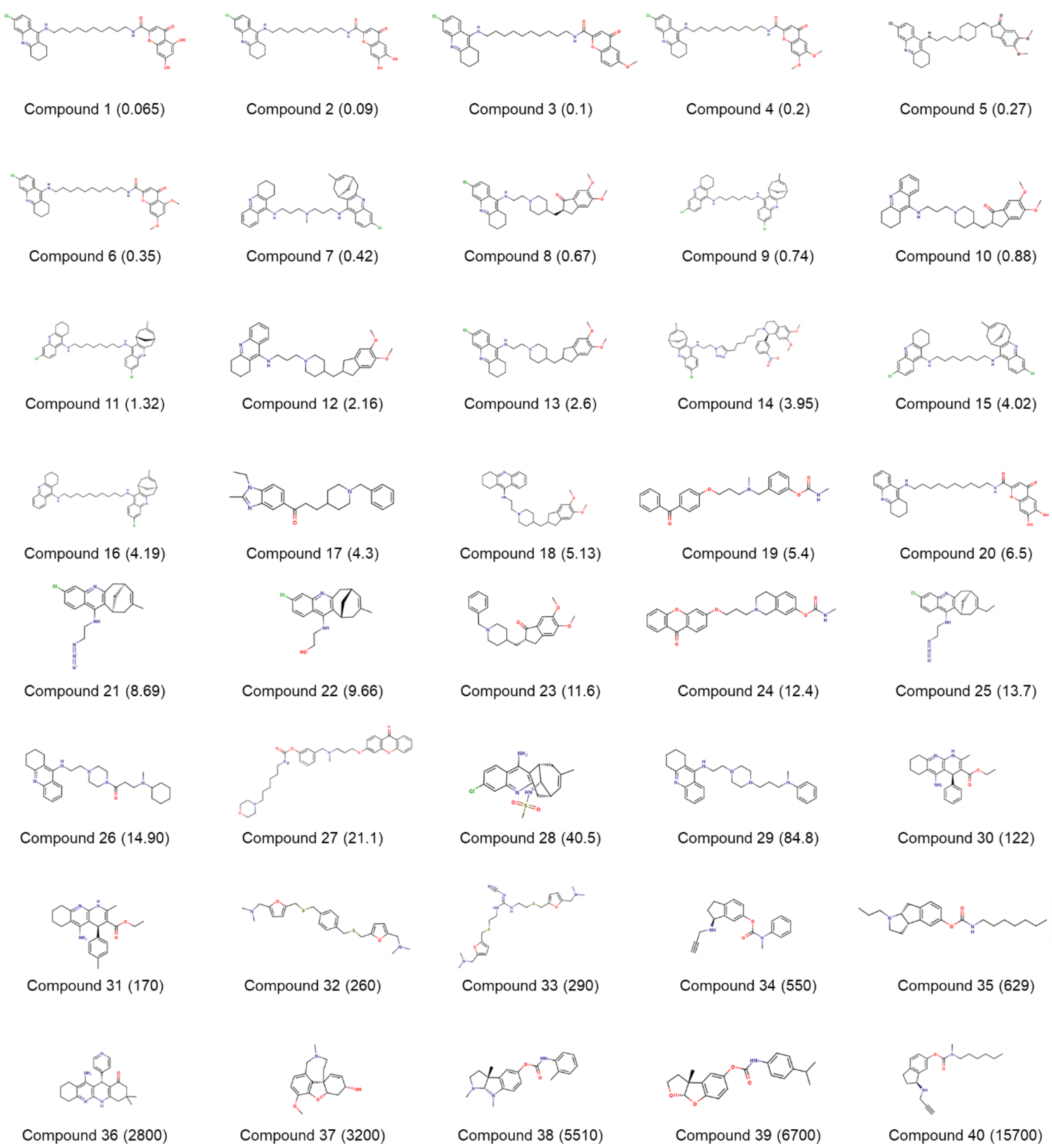

Figure S1. 2D structures of 40 compounds in the test set. The inhibitory activity value. (IC_{50}) for each compound was shown in nM.

Table S1. Experimental and estimated IC_{50} values of the test set using the best hypothesis, Hypo 1.

Compound no.	Experimental IC_{50} (nM)	Estimated IC_{50} (nM)	Error[a]	Experimental scale[b]	Estimated scale[b]	Fit value[c]
1	0.065	0.25	+3.89	++++	++++	7.99
2	0.09	0.26	+2.91	++++	++++	7.97
3	0.1	0.24	+2.45	++++	++++	8.00
4	0.2	0.25	+1.26	++++	++++	7.99
5	0.27	0.32	+1.19	++++	++++	7.88
6	0.35	0.25	-1.43	++++	++++	8.00
7	0.42	1.07	+2.55	++++	++++	7.36
8	0.67	0.79	+1.18	++++	++++	7.49
9	0.74	0.61	-1.21	++++	++++	7.60
10	0.88	0.38	-2.33	++++	++++	7.81
11	1.32	2.96	2.24	++++	++++	6.92
12	2.16	0.56	-3.86	++++	++++	7.64
13	2.6	1.23	-2.12	++++	++++	7.30
14	3.95	0.30	-13.05	++++	++++	7.91
15	4.02	3.11	-1.29	++++	++++	6.90
16	4.19	0.97	-4.32	++++	++++	7.40
17	4.3	0.35	-12.44	++++	++++	7.85
18	5.13	0.73	-7.08	++++	++++	7.53
19	5.4	0.43	-12.47	++++	++++	7.75
20	6.5	0.26	-24.97	++++	++++	7.97
21	8.69	3.57	-3.14	++++	++++	6.95
22	9.66	272.87	+28.25	++++	++	4.95
23	11.6	2.11	-5.49	++++	++++	7.06
24	12.4	1.31	-9.48	++++	++++	7.27
25	13.7	1.59	-8.62	++++	++++	7.19
26	14.9	0.35	-43.15	++++	++++	7.85
27	21.1	0.43	-48.80	+++	++++	7.75
28	40.5	152.05	+3.75	+++	+++	5.21
29	84.8	26.40	-3.21	+++	+++	5.97
30	122	696.35	+5.71	+++	++	4.55
31	170	1654.59	+9.73	+++	++	4.17

32	260	12.21	-21.30	++	++++	6.30
33	290	9.52	-30.46	++	++++	6.41
34	550	6060.45	+11.02	++	+	3.61
35	629	15216.87	+24.19	++	+	3.21
36	2,800	39854.87	+14.23	+	+	2.79
37	3,200	246.40	-12.99	+	++	5.00
38	5,510	208709.79	+37.88	+	+	2.07
39	6,700	263919.66	+39.39	+	+	1.97
40	15,700	21768.08	+1.39	+	+	3.05

[a]Error: Difference between the experimental and estimated IC_{50} values. Positive value indicates that the estimated value is higher than the experimental value; negative value indicates that the estimated value is lower than the experimental value.

[b]Activity scale: ++++, IC_{50} < 20 nM (most active); +++, 20 ≤ IC_{50} < 200 nM (active); ++, 200 ≤ IC_{50} <2000nM (moderately active); +, IC_{50} ≥ 2000 nM (inactive).

[c]Fit value represents how well the pharmacophoric features in the hypothesis overlap the chemical features in the compound.

Table S2. The physico-chemical properties for the obtained hit compounds in this study.

	IUPAC name	Molecular formula	Molecular weight	ALogP	Molecular polar surface area	No. of hydrogen bond acceptors	No. of hydrogen bond donors	No. of rotatable bonds
Hit 1	(5R)-4-[4-(benzyloxy)-2-methylbenzoyl]-3-hydroxy-1-(2-methoxyethyl)-5-(pyridin-4-yl)-2,5-dihydro-1H-pyrrol-2-one	$C_{27}H_{26}N_2O_5$	458.506	3.157	88.96	6	1	9
Hit 2	(5S)-4-[4-(benzyloxy)-3-methylbenzoyl]-1-[3-(dimethylamino)propyl]-3-hydroxy-5-(pyridin-4-yl)-2,5-dihydro-1H-pyrrol-2-one	$C_{29}H_{31}N_3O_4$	485.574	3.489	82.97	6	1	10
Hit 3	(5S)-3-hydroxy-4-[2-methyl-4-(2-methylpropoxy)benzoyl]-1-[3-(morpholin-4-yl)propyl]-5-(pyridin-4-yl)-2,5-dihydro-1H-pyrrol-2-one	$C_{28}H_{35}N_3O_5$	493.595	2.785	92.2	7	1	10
Hit 4	(5S)-5-(3-ethoxyphenyl)-4-(furan-2-carbonyl)-3-hydroxy-1-[2-(morpholin-4-yl)ethyl]-2,5-dihydro-1H-pyrrol-2-one	$C_{23}H_{26}N_2O_6$	426.462	1.939	92.45	6	1	8
